# Memory Loss and Missteps: Investigating Fall Risks in Alzheimer’s and Dementia Patients

**DOI:** 10.20900/agmr20240005

**Published:** 2024-09-30

**Authors:** Asmaa Namoos, Nicholas Thomson, Sarah Bradley, Amanda Rudderman, Michel Aboutanos

**Affiliations:** Department of Surgery, Injury and Violence Prevention Program, School of Medicine, Medical Center West Hospital, Virginia Commonwealth University (VCU), 1200 E Broad Steet, Richmond, VA 23219, USA

**Keywords:** Alzheimer’s disease, dementia, falls, older adults, traumatic brain injury (TBI), epidemiology, prevention

## Abstract

**Background:**

Degenerative diseases such as Alzheimer’s disease and dementia are significant health concerns among older adults in the United States, contributing substantially to the high incidence of falls in this population. This study aims to investigate the incidence and prevalence of falls among older adults diagnosed with Alzheimer’s disease and dementia and explore the association between these conditions and the occurrence of traumatic brain injuries (TBIs).

**Methods:**

A retrospective cohort study was conducted using data from 17,000 older adults aged 65 and above, arrived at the hospital with fall related injuries, obtained from the TriNetX network at Virginia Commonwealth University Health System (VCUHS) between January 1, 2019, and December 31, 2023. Data included demographic information, diagnosis codes (ICD-10), and details on falls, Alzheimer’s disease, dementia, and TBIs. Descriptive statistics and logistic regression analyses were performed using TriNetX analytical tools.

**Results:**

Older adults with Alzheimer’s disease (incidence proportion: 3.11%, prevalence: 4.81%) and dementia (incidence proportion: 12.46%, prevalence: 17.06%) had a significantly higher incidence of falls compared to those without these conditions. Females showed a slightly higher incidence of falls than males. Logistic regression analysis indicated that patients with Alzheimer’s disease had a reduced risk of TBIs (OR = 0.765, 95% CI: 0.588–0.996, *p* = 0.047), while those with unspecified dementia had an increased risk (OR = 1.161, 95% CI: 1.002–1.346, *p* = 0.047).

**Conclusions:**

Our study reveals a higher risk of falls and traumatic brain injuries (TBIs) in older adults with dementia compared to those with Alzheimer’s disease. These findings underscore the need for targeted fall prevention strategies and educational programs for caregivers.

## BACKGROUND

Annually, one in four older adults experiences a fall, leading to approximately 3 million emergency department visits [1,2]. The risk of falls escalates with advancing age, with individuals aged 85 and older being at the highest risk [3,4]. Degenerative diseases, including Alzheimer’s disease and dementia, are significant contributors to the high incidence of falls among older adults [5–7].

Cognitive impairments associated with Alzheimer’s disease affect an individual’s ability to recognize hazards and respond appropriately to environmental challenges. Additionally, motor deficits and balance issues commonly seen in Alzheimer’s patients further elevate the risk of falling [8]. Studies have shown that individuals with Alzheimer’s disease are twice as likely to experience falls compared to those without the disease [9].

Similarly, dementia, which encompasses a range of cognitive impairments including Alzheimer’s disease, is strongly linked to an increased risk of falls [10]. Dementia and Alzheimer’s affects approximately 6.9 million adults aged 65 and older in the United States [11]. The cognitive deficits associated with dementia, including impaired executive function and attention, can significantly increase the risk of major nervous system injuries [12]. These deficits hinder an individual’s ability to navigate their environment safely, making them more susceptible to falls and other accidents that can result in severe injuries to the nervous system, such as Traumatic Brain Injuries (TBI) [13,14].

The outcomes of TBIs in older adults can be devastating, leading to prolonged hospitalizations, increased morbidity, and higher mortality rates [15]. The CDC reports that falls are the leading cause of TBI in older adults, accounting for approximately 60% of all TBI-related hospitalizations in this age group [16]. The economic burden of neurodegenerative and cognitive disorders is staggering on both a global and national scale. The global cost of traumatic brain injury (TBI) is estimated to be $400 billion annually, which is 0.5% of the world’s annual output [17]. The total lifetime cost of care for a person living with dementia is estimated at almost $400,000, with seventy percent of these costs borne by family caregivers [18]. Alzheimer’s disease alone costs the U.S. economy an estimated $321 billion in 2022, in addition to approximately $271 billion in unpaid caregiving [19]. Hence, comprehending the relationship between falls, degenerative diseases, and TBIs is essential for formulating effective prevention and intervention strategies to reduce these risks.

The primary aim of this study is to investigate the incidence and prevalence of falls among older adults diagnosed with Alzheimer’s disease and dementia over a five-year period. Additionally, the study explores the risk of falls between Alzheimer’s disease and dementia and the occurrence of traumatic brain injuries (TBIs), specifically diffuse TBIs with a loss of consciousness lasting between 6 and 24 hours.

## METHODS

### Study Design

This retrospective cohort study utilizes a dataset of 17,000 older adults aged 65 and above who experienced falls, as identified through their medical records. The study period spans from January 1, 2019, to December 31, 2023. The cohort included patients with a history of Alzheimer’s disease (AD), dementia, and traumatic brain injuries (TBIs), specifically those with diffuse TBI and a loss of consciousness lasting between 6 and 24 hours. This study categorized patients into three distinct groups: (1) patients with dementia and a history of falls, (2) patients with Alzheimer’s disease and a history of falls, and (3) patients without either neurocognitive condition but with a history of falls. These groups were defined using ICD-10 diagnostic codes and clinical history recorded in the electronic health records. Fall history was identified through fall-related ICD-10 codes, and all neurocognitive diagnoses were confirmed prior to inclusion in the analysis. This classification enabled a comprehensive evaluation of fall risk across different levels of cognitive impairment.

### Data Collection

Data were extracted from the electronic health records (EHRs) available in the TriNetX network at Virginia Commonwealth University Health System (VCUHS). The dataset includes detailed demographic information (age, sex, race, and ethnicity), diagnosis codes (ICD-10), and information on fall incidents, Alzheimer’s disease, dementia, and TBIs. Additional data on comorbidities, medications, and previous fall history were also included to account for potential confounders.

### Data Source

TriNetX is a global health research network that connects healthcare organizations and life sciences companies, utilizing real-world data to accelerate clinical research and the development of new therapies. Through its platform, TriNetX provides access to aggregated and anonymized patient data from electronic health records, enabling researchers to conduct observational studies, optimize clinical trials, and generate real-world evidence for healthcare advancements [20].

#### Incidence and prevalence calculation

Incidence proportion in TriNetX is the rate of new or first-time cases within a cohort during a given time window. Patients are included in the denominator if their records overlap the time window, and they have no prior diagnosis of the event of interest within a set lookback period. The incidence rate accounts for the time at risk, calculated by multiplying the incidence proportion denominator by the number of days in the time window. Both the numerator and denominator align with those used for the incidence proportion. Prevalence reflects all cases within or before the time window. The prevalence denominator includes patients whose records overlap the time window, and the numerator includes those with a relevant diagnosis either before or during the time window. These calculations follow TriNetX’s established framework for analyzing real-world EHR-based data [21].

The validity of these measures was ensured by utilizing standardized ICD-10 codes for fall-related injuries, Alzheimer’s disease, dementia, and TBIs. The following ICD-10 codes were used [22] (Table 1):

ICD-10 codes for falls, Alzheimer’s disease, dementia, and TBIs have been extensively validated and are reliable for identifying these medical conditions in clinical datasets. The TriNetX platform provided a comprehensive capture of medical records across healthcare settings, improving the reliability of the data.

### Handling of Multiple Records and Missing Data

The unit of analysis was the patient. In cases where multiple encounters were recorded for a single patient, only the first fall-related event was used to calculate incidence. Subsequent falls were included in the prevalence analysis but not counted as new incidents unless the fall occurred more than six months after the first event.

#### For missing data

The TriNetX platform aggregates real-world data from electronic health records (EHRs) across healthcare organizations, ensuring that all necessary patient data are complete and de-identified. TriNetX’s federated data network maintains rigorous data governance protocols, minimizing the occurrence of missing data. Therefore, for this analysis, there were no missing data for key variables such as diagnosis codes, demographic characteristics, or clinical outcomes.

### Exclusion Criteria for New Cases

To ensure that the data captured only new cases of Alzheimer’s disease, dementia, and TBIs during the study period, any patient with a preexisting ICD-10 code for these diagnoses prior to the study period was excluded from the incidence calculations. Prevalence was calculated by including all patients with a recorded diagnosis of these conditions during the study period, regardless of prior history.

### Statistical Analysis

Our analysis was conducted on the TriNetX platform. Descriptive statistics were used to summarize demographic characteristics of the study population. Continuous variables, such as age, were expressed as means and standard deviations, while categorical variables, including sex, race, and ethnicity, were reported as frequencies and percentages.

Univariate analyses were performed using Chi-square tests for categorical variables and T-tests for continuous variables. We calculated the incidence and prevalence of falls, Alzheimer’s disease, dementia, and TBIs, stratified by demographic variables such as age, sex, race, and ethnicity. Logistic regression analyses were conducted to assess associations between Alzheimer’s disease, dementia, and the risk of TBIs. Both univariate and multivariable models were used, with adjusted odds ratios (ORs) and 95% confidence intervals (CIs) reported, accounting for potential confounders such as age, sex, comorbidities, medications, and previous fall history.

### Software and Tools

All analyses were conducted using the TriNetX platform’s analytical tools. These tools enabled processing of large datasets and provided robust statistical evaluations and visualizations to support epidemiological analysis.

## RESULTS

### Demographic Characteristics

The dataset included 17,000 older adults aged 65 and above, who had experienced falls, spanning a wide range of ages from 65 to 89 years, with an average age of 78 years and a standard deviation of 8 years. The demographic breakdown revealed a predominant female representation at approximately 59.29%, compared to 40.70% male, and a minimal percentage (0.05%) with unspecified sex. Ethnically, the majority were non-Hispanic or Latino (96.64%), with a small representation of Hispanic or Latino individuals (0.52%). Racial demographics showed a significant portion of White individuals (61.70%), followed by Black or African American (33.58%). Other racial categories included Asian (0.52%), American Indian or Alaskan Native (0.11%), and Native Hawaiian or Other Pacific Islander (0.05%), with a small percentage of patients identified as other races (2.58%) or unknown (1.58%) (Table 2).

The demographic distribution of Alzheimer’s disease among older adults over a five-year period revealed notable trends in incidence and prevalence. Incidence began at 1.85% for those aged 65–69 and increased to 6.83% for individuals aged 85 and older. Prevalence similarly rose from 2.57% to 11.83% across the same age groups.

Females had a slightly higher incidence proportion (3.41%) and prevalence (5.02%) compared to males, who had an incidence proportion of 2.85% and a prevalence of 4.49%. The incidence rates per person-day were 0.0000267 for females and 0.0000240 for males. Asian individuals had the highest incidence proportion at 12.5%, with a corresponding prevalence and incidence rate of 0.0001039 cases per person-day. White individuals had an incidence proportion of 3.14%, a prevalence of 4.74%, and an incidence rate of 0.0000257 cases per person-day.

Hispanic or Latino individuals exhibited a significantly higher incidence proportion at 16.67%, with the same prevalence and an incidence rate of 0.0003091 cases per person-day. Non-Hispanic or Latino individuals showed an incidence proportion of 3.07%, a prevalence of 4.83%, and an incidence rate of 0.0000246 cases per person-day (Table 3).

The analysis of falls among older adults with dementia revealed significant demographic patterns. Among age groups, individuals aged 85 and older exhibited the highest incidence proportion at 28.08%, with a prevalence of 37.87% and an incidence rate of 0.0003058 cases per person-day. In contrast, those aged 65–69 years had the lowest incidence proportion at 7.58%, with a prevalence of 10.29% and an incidence rate of 0.0000585 cases per person-day.

Females had an incidence proportion of 12.28%, with a prevalence of 17.20% and an incidence rate of 0.0000989 cases per person-day, while males had a slightly higher incidence proportion at 12.75%, with a prevalence of 16.82% and an incidence rate of 0.0001107 cases per person-day.

Individuals identified as Black or African American had an incidence proportion of 13.92% and a prevalence of 19.87%, with an incidence rate of 0.0001095 cases per person-day, while White individuals showed an incidence proportion of 11.80%, a prevalence of 15.66%, and an incidence rate of 0.0000993 cases per person-day. Asian individuals had an incidence proportion and prevalence of 12.5%, with an incidence rate of 0.0001109 cases per person-day.

Hispanic or Latino individuals had a notably high incidence proportion of 33.33%, with the same prevalence and an incidence rate of 0.0006996 cases per person-day. Non-Hispanic or Latino individuals had an incidence proportion of 12.43%, a prevalence of 17.19%, and an incidence rate of 0.0001024 cases per person-day (Table 4).

Alzheimer’s Disease Vs Dementia—5 Year Fall Analysis. The five-year fall analysis highlights differences in the incidence and prevalence of falls among older adults with Alzheimer’s disease and dementia. Alzheimer’s disease shows an incidence proportion of 3.11% and a prevalence of 4.81%, with an incidence rate of 0.0000251 cases per person-day related to falls. In contrast, dementia exhibits a much higher incidence proportion at 12.46% and a prevalence of 17.06%, with an incidence rate of 0.0001033 cases per person-day related to falls.

The incidence proportion of falls in individuals with dementia is approximately four times higher than in those with Alzheimer’s disease (12.46% vs. 3.11%). Similarly, the prevalence of falls is about 3.5 times higher in individuals with dementia compared to those with Alzheimer’s disease (17.06% vs. 4.81%).

The logistic regression analysis revealed significant differences in the relationship between Alzheimer’s disease, dementia, and the occurrence of diffuse traumatic brain injury (TBI) with a loss of consciousness lasting between 6 and 24 hours in patients aged 65 years and older. Among patients who experienced a fall, those with Alzheimer’s disease (390 out of 910) were 23.5% less likely to suffer from this type of brain injury compared to patients without Alzheimer’s or dementia, with a crude odds ratio of 0.765 (95% CI: 0.588–0.996, *p* = 0.047), and an adjusted odds ratio of 0.755 (95% CI: 0.576–0.990). In contrast, patients with dementia (1210 out of 3080) were 16.1% more likely to experience diffuse traumatic brain injury compared to the same reference group, with a crude odds ratio of 1.161 (95% CI: 1.002–1.346, *p* = 0.047), and an adjusted odds ratio of 1.158 (95% CI: 1.002–1.339). The p-values (both 0.047) are very close to the conventional threshold for statistical significance (*p* = 0.05), indicating marginal significance. Notably, dementia showed a stronger association with the risk of TBI compared to Alzheimer’s disease.

## DISCUSSION

A retrospective study provided significant insights into the incidence and prevalence of falls among older adults diagnosed with Alzheimer’s disease and dementia, and their association with traumatic brain injuries (TBIs). The results underscored the heightened vulnerability of this population to falls and the severe injuries that could arise, such as TBIs.

Our study supports the findings by the CDC, indicating that the risk of falls increases with advanced age [23]. Additionally, our study confirmed that this trend was also evident among Alzheimer’s and dementia patients who came to the emergency department with fall-related injuries [3,4]. The female cohort had a slightly higher incidence of falls than the male cohort. This finding was consistent with previous research indicating that older women were more prone to falls with injury due to factors such as lower bone density and muscle mass [24,25]. Hewson et al. reported high fall rates among Asians compared with other racial groups [26].

Our study confirmed these findings, indicating that the incidence of falls among Alzheimer’s and dementia patients varied significantly across racial and ethnic groups. Asian individuals exhibited the highest incidence of falls related to Alzheimer’s disease, while Black or African American individuals showed the highest incidence of falls related to dementia. These demographic alignments served as a key motivator for us to develop risk-level assessment tools (low, medium, and high risk) in the neurological assessment section to enroll cognitive disorder patients in targeted interventions. By categorizing patients based on their risk levels, we implemented caregiver intervention strategies to mitigate fall risks effectively at the family level.

Having analyzed the demographic data on the incidence and prevalence of falls among older adults with Alzheimer’s disease and dementia, we shifted our focus to contextualizing these findings within the broader landscape of existing research. The analysis revealed that individuals with dementia exhibited a fourfold higher risk of falls compared to those with Alzheimer’s disease. This heightened risk could be attributed to greater cognitive and physical impairments in dementia patients, including more severe motor dysfunction, impaired spatial awareness, and greater behavioral symptoms like agitation and wandering, which increase fall risk [6]. While previous studies have highlighted fall risks in dementia, our study adds to the body of literature by quantifying this risk and focusing on emergency department populations.

The people with dementia were reported to have a substantially higher risk of falls and fall-related injuries [10,12,13]. For instance, studies have shown that seniors with dementia were up to three times more likely to suffer hip fractures compared to those without cognitive impairments, highlighting the profound impact of cognitive decline on physical stability [27].

While TBI is associated with cognitive diseases [28], the association between Alzheimer’s disease and a reduced risk of TBIs observed in this study was unexpected. The logistic regression analysis compared patients with Alzheimer’s disease and dementia to a control group of patients without either condition but with a history of falls. This finding could have been influenced by several factors, including the severity of Alzheimer’s disease and the level of care provided to these patients, which may have included measures to prevent severe injuries. However, this area requires further investigation to understand the underlying mechanisms and to validate these results.

Conversely, the study found that individuals with unspecified dementia had an increased risk of TBIs, highlighting the need for targeted interventions in this group. The cognitive and physical impairments associated with dementia, such as poor coordination and balance, likely contributed to this elevated risk. This finding emphasized the importance of comprehensive fall prevention programs that address both cognitive and physical health in dementia patients.

The use of ICD-10 codes to identify Alzheimer’s disease, dementia, falls, and traumatic brain injuries (TBIs) in this study provides a high level of validity and reliability in our findings. ICD-10 codes are widely accepted in clinical research and have been extensively validated for identifying medical conditions across various healthcare settings [29]. The accuracy of these codes ensures consistent and standardized data collection, which is crucial for a retrospective study like ours. However, it is important to acknowledge that while ICD-10 codes offer a reliable method of diagnosis, they may not capture the full complexity of the conditions, such as the severity of cognitive decline or subtle variations in fall circumstances. Future studies could benefit from incorporating additional clinical data to complement the information obtained from ICD-10 coding.

The findings from our study could significantly enhance the development of risk assessment tools for older adults with cognitive impairments. By identifying that individuals with dementia have a markedly higher risk of falls compared to those with Alzheimer’s disease, we revised our scoring system to classify fall risk based on comorbidities, ranking diseases according to their impact on fall risk. This approach enabled us to develop a comprehensive and accurate tool that considers the presence and severity of comorbidities, allowing clinicians to better assess and prioritize interventions. The focus on caregiver-targeted interventions has facilitated the implementation of tailored education programs, further improving patient safety and outcomes. By integrating these strategies, we ensure that the refined assessment tools are evidence-based, valid, and clinically relevant, ultimately enhancing fall prevention efforts for vulnerable populations.

Strengths. The study boasts several strengths. The large sample size of 17,000 older adults provides robust statistical power, enhancing the reliability of our findings. However, while statistical significance can be achieved with large samples, it is important to note that it does not always equate to clinical significance. The real-world applicability of these findings depends on their ability to drive meaningful improvements in patient care, particularly when translating statistical differences into effective interventions. The use of standardized ICD-10 codes ensures consistent and accurate identification of medical conditions. Furthermore, the comprehensive demographic and clinical data allow for detailed analyses and stratification by various factors, enriching our understanding of fall risks in older adults with cognitive impairments.

## LIMITATIONS

One key limitation of our study is its retrospective nature, which relies on previously collected data and may be subject to incomplete or inaccurate electronic health records (EHR). Additionally, the use of de-identified data prevents us from conducting follow-up assessments or validating the accuracy of recorded diagnoses and fall incidents. Furthermore, the study’s setting within the Virginia Commonwealth University Health System (VCUHS) may limit the generalizability of the findings to other populations or healthcare settings, as practices and patient demographics can vary widely.

## CONCLUSION

In conclusion, our study highlights the significant risk of falls and traumatic brain injuries (TBIs) among older adults with Alzheimer’s disease and dementia. The findings underscore the markedly higher incidence and prevalence of falls in individuals with dementia compared to those with Alzheimer’s disease. This differential risk emphasizes the need for targeted fall prevention strategies and educational programs tailored to caregivers of dementia patients. By refining risk assessment tools and implementing evidence-based interventions, researchers and program interventionists can improve patient safety and outcomes, ultimately enhancing the quality of life for older adults with cognitive impairments. Our results provide a solid foundation for future research aimed at developing more effective fall prevention programs and exploring the underlying mechanisms of fall-related injuries in this vulnerable population.

## Figures and Tables

**Table 1.** ICD-10 codes for Alzheimer’s disease, dementia, falls, and traumatic brain injuries (TBIs).

Condition	ICD-10 Code	Description

Alzheimer’s Disease	G30.0	Alzheimer’s disease with early onset
	G30.1	Alzheimer’s disease with late onset
	G30.8	Other Alzheimer’s disease
	G30.9	Alzheimer’s disease, unspecified

Dementia	F01.0	Vascular dementia with acute onset
	F01.1	Multi-infarct dementia
	F01.2	Vascular dementia with subcortical damage
	F01.3	Mixed cortical and subcortical vascular dementia
	F01.8	Other vascular dementia
	F01.9	Vascular dementia, unspecified
	F02.80	Dementia in other diseases, without behavioral disturbance
	F02.81	Dementia in other diseases, with behavioral disturbance
	F03	Unspecified dementia
	G31.0	Frontotemporal dementia
	G31.83	Dementia with Lewy bodies

Falls	W00	Fall due to ice and snow
	W01	Fall on same level from slipping, tripping, and stumbling
	W02	Fall involving ice-skates, skis, roller-skates, and skateboards
	W10	Fall on and from stairs and steps
	W19	Unspecified fall

Traumatic Brain Injuries (TBIs)	S06.0	Concussion
	S06.1	Traumatic cerebral edema
	S06.2	Diffuse traumatic brain injury
	S06.3	Focal traumatic brain injury
	S06.4	Epidural hemorrhage
	S06.5	Traumatic subdural hemorrhage
	S06.6	Traumatic subarachnoid hemorrhage
	S06.8	Other specified intracranial injuries
	S06.9	Unspecified intracranial injury

**Table 2.** Demographic characteristics of older adults with fall-related injuries.

Variable	Category	Percentage	Count

Total Patients		100%	17,000

Age	Mean = 78, SD = 8		

Sex	Female	59.29%	10,079
	Male	40.70%	6919
	Unknown	0.05%	9

Ethnicity	Not Hispanic or Latino	96.64%	16,429
	Hispanic or Latino	0.52%	88
	Unknown Ethnicity	2.88%	490

Race	American Indian or Alaskan Native	0.11%	19
	Asian	0.52%	88
	Black or African American	33.58%	5709
	Native Hawaiian or Other Pacific Islander	0.05%	9
	Other Race	2.58%	439
	Unknown Race	1.58%	269
	White	61.70%	10,489

**Table 3.** Incidence and prevalence of Alzheimer’s disease in older adults with fall-related injuries over five years.

Demographic	Category	Incidence Proportion	Prevalence	Incidence Rate (cases/person-day)

Age	65–69 years	1.85%	2.57%	0.000014
	70–74 years	3.16%	3.92%	0.000024
	75–79 years	4.02%	6.37%	0.000033
	80–84 years	6.49%	10.00%	0.000056
	85 and older	6.83%	11.83%	0.000069

Sex	Female	3.41%	5.02%	0.0000267
	Male	2.85%	4.49%	0.0000240

Ethnicity	Hispanic or Latino	16.67%	16.67%	0.0003091
	Not Hispanic or Latino	3.07%	4.83%	0.0000246
	Unknown Ethnicity	0.05%	0.05%	0.0000468

Race	White	3.14%	4.74%	0.0000257
	Black or African American	3.08%	4.97%	0.0000234
	Asian	12.5%	12.5%	0.0001039
	Other Race	5.56%	5.56%	0.0000539
	Unknown Race	4.35%	8.70%	0.0000546
	American Indian or Alaska Native	0.0%	0.0%	0.0
	Native Hawaiian or Other Pacific Islander	0.0%	0.0%	0.0

**Table 4.** Incidence and prevalence of Alzheimer’s disease in older adults with fall-related injuries over five years.

Demographic	Category	Incidence Proportion	Prevalence	Incidence Rate (cases/person-day)

Age	65–69 years	7.58%	10.29%	0.0000585
	70–74 years	11.43%	14.90%	0.0000904
	75–79 years	16.15%	21.08%	0.0001410
	80–84 years	22.07%	29.38%	0.0002025
	85 and older	28.08%	37.87%	0.0003058

Sex	Female	12.28%	17.20%	0.0000989
	Male	12.75%	16.82%	0.0001107

Ethnicity	Hispanic or Latino	33.33%	33.33%	0.0006996
	Not Hispanic or Latino	12.43%	17.19%	0.0001024
	Unknown Ethnicity	0.1%	0.12%	0.0000963

Race	White	11.80%	15.66%	0.0000993
	Black or African American	13.92%	19.87%	0.0001095
	Asian	12.5%	12.5%	0.0001109
	American Indian or Alaska Native	0.5%	0.5%	0.0004033
	Native Hawaiian or Other Pacific Islander	0.0%	0.0%	0.0
	Other Race	11.76%	16.67%	0.0001133
	Unknown Race	13.64%	17.39%	0.0001744

## Data Availability

The data that support the findings of this study are available upon reasonable request. Interested researchers can obtain access to the data by submitting a formal request to the corresponding author at Asmaa.namoos@vcuhealth.org. The data is not publicly available due to privacy or ethical restrictions.
